# Aging in (a meaningful) place – appropriateness and feasibility of Outdoor Reablement in a rural Arctic setting

**DOI:** 10.1186/s12913-024-12031-7

**Published:** 2024-12-18

**Authors:** Marianne Eliassen, Trude Anita Hartviksen, Solrun Holm, Bodil Anita Sørensen, Magnus Zingmark

**Affiliations:** 1 Department of Health and Care Sciences, UiT, The Artic University of Norway, Tromsø, 9037 Norway; 2 Centre for Care Sciences North, UiT, The Arctic University of Norway, Tromsø, 9037 Norway; 3 Leknes, Norway; 4 Department for Community Medicine and Rehabilitation, Umeå University, Umeå, Sweden; 5 Municipality of Östersund, Health and Social Care Administration, Östersund, Sweden

**Keywords:** Age-friendly cities and communities, Aging in place, Outdoor environment, Co-design, Health care services, Rural Arctic community

## Abstract

**Background:**

As populations age in the Western world, interventions aiming for ‘aging in place’, such as reablement, have gained prominence. Reablement programs have focused on enabling older people to maintain independence in their home environment. However, while a growing body of research points to the considerable benefits of engaging in outdoor environments, reablement rarely addresses outdoor activities. People living in rural Arctic areas often tend to have strong cultural, social, and emotional attachments to outdoor places, emphasizing the outdoors as a meaningful arena for engagement. Concurrently, rural Arctic communities face unique obstacles in facilitating outdoor activities, such as geographic isolation, limited access to services, harsh climate conditions, and seasonal variations. Recognizing these challenges, our study sought to tailor an outdoor reablement model that is appropriate and feasible for the context of a rural Arctic setting.

**Methods:**

The study design was inspired by a co-design methodology, incorporating data creation through workshops, focus groups, and individual interviews conducted over an eleven-month period. Three municipalities in rural Arctic Norway were involved, with a total of 35 participants, including older people receiving reablement services and healthcare professionals. A socioecological theory supported the thematic data analysis.

**Results:**

The study yielded experiences that generated a comprehensive model for implementing outdoor reablement that meet the specific needs that the participants experienced in the rural Arctic setting. The model includes the individual level, accounting for physical and mental functioning; the organizational level, necessitating access to aids and equipment and cross-sectorial collaboration; and the environmental level, adapting to climatic, seasonal, and geographic challenges.

**Conclusion:**

This study contributes with knowledge that broadens the scope of reablement as an initiative to support aging in place to include outdoor environments. The tailored outdoor reablement model developed in this study addresses the complexity of aging in place in rural Arctic settings. The study underscores the importance of context-specific strategies that support older people in maintaining a healthy and meaningful life through active engagement with the outdoors.

**Supplementary Information:**

The online version contains supplementary material available at 10.1186/s12913-024-12031-7.

## Background

In response to the global demographic shift toward an aging population, there has been a surge of international interest in policies that support “aging in place” [1], a concept that was first promoted by the Organisation for Economic Co-operation and Development (OECD) in 1994 [2]. Although people can remain healthy and independent well into old age, aging does include changes in biological, social, or cognitive processes, e.g. loss of muscle strength, decreased bone mass density, reduced vision and hearing abilities, decreased participation in societal activities, increased risk of social isolation, and reduced cognitive capacity [3]. Reduced ability to perform activities that are perceived as meaningful may pose health challenges for the older person [4] and difficulties with managing life in their home context. Aging in place strategies aim to support older people to remain living in their own homes, retaining connection with their social network and maintain engagement in daily activities [1]. The aging in place strategy is considered not only for cost effectiveness [5] but also as a symbol of human rights, independence, and autonomy for older people [1].

However, the definition of aging in place is not without unambiguity. While some interpretations focus on the ability to live in one’s own *home* for as long as possible, others highlight the importance of remaining living within the *community* [6]. Thus, aging in place may encompass more than the mere desire to stay at home; it also involves an attachment to social connections, security, familiarity, and a sense of identity associated with places [7].

While strategies and interventions that support aging in urban areas are well studied [8], rural settings remain underexplored [9]. This is problematic, as the proportion of older people is increasing more rapidly in rural communities than in urban areas [10]. A scoping review [9] revealed that interventions in rural areas were predominantly health- or disease-oriented, often neglecting environmental and contextual factors that can contribute to an active life, such as transportation services and environmental adjustments.

The rural Arctic environment in northern Norway, characterized by seasonal variations in climate, along with vast distances and underdeveloped transportation systems, presents unique challenges for promoting aging in place. Environmental factors, such as long and dark snowy winters and remote community facilities, are associated with the occurrence of frailty [11] and may force frail older people toward urbanization [12], thereby impeding aging in place. Hence, the interplay between an individual’s physical, mental, and social resources and these contextual challenges is especially demanding in the rural Arctic [11].

Traditionally, people living in rural Arctic areas in Norway have been living in close relationships with nature and seasonal variations, as fishing and harvesting from nature have been important for both labor and leisure [13, 14]. People who reside in areas where they engage closely with nature often find significant meaning from their interaction with the natural environment and outdoor settings. Participation in outdoor environments has been shown to impact positively on social participation [8, 15], reduce loneliness [16], and improve mental health [17]. A recent review of nature-based interventions concludes that there are positive effects on circulatory, neurological, endocrine, muscular, and respiratory parameters [18], and as little as short walks outdoors have been shown to prevent physical decline among older people [19]. Maintaining participation in such environments is crucial for preserving a sense of meaningful living into old age [20–23]. In this context, understanding the cultural attachment to places that prevail for many people living in the rural Arctic is foundational for how to achieve meaningful aging in such areas [20].

The Norwegian welfare services are founded on the principle of universalism, ensuring equitable access to health and welfare services for all citizens [24–26]. Consequently, older people in rural Arctic communities should have the same opportunities to access services that support aging in place as those in urban areas. Therefore, environmental strategies that promote healthy and active aging in the rural Arctic can address societal inequities by enhancing social engagement and participation, which are crucial for the well-being and longevity of older people [1].

The implementation of aging in place strategies in rural Arctic communities necessitates a multisectoral and socioecological approach that comprehensively meets the needs of individuals and the communities they inhabit [9, 27]. Person-centered services cannot be based solely on knowledge about individual features, as the planning and design of services must incorporate insights into the physical and social environments where individuals reside. However, contextual adaptations are only sporadically referenced in research [28], and descriptions of the specific challenges in the rural Arctic context are sparse in Nordic national governmental papers [29], posing challenges for the adoption and implementation of novel interventions in such contexts.

Aging in place strategies require initiatives that aim to prevent or mitigate functional decline and loss of independence and promote older peoples’ participation in daily activities that the person considers meaningful [30]. Reablement is a team-based, person-centered, holistic intervention designed to enhance functioning and support independence in meaningful daily activities at one’s place of residence [31]. The ideology of aging in place is foundational to reablement, as it is intended to increase functioning and social participation within the local community [31, 32]. However, existing research suggests that reablement has focused predominantly on home-based activities, such as personal activities of daily living (PADL) and indoor mobility, often overlooking outdoor activities and community engagement [33–35].

In a recent study, we developed an outdoor reablement model to integrate outdoor activities into the reablement process, recognizing the significance of place in the lives of older people [20]. The organizational framework for the model was based on descriptions of reablement services [31] and included home visits by an interprofessional team, consisting of a physiotherapist (PT), occupational therapist (OT), registered nurse (RN), and associate nurse (AN). An initial assessment identified the user’s individual goals and functional level, and a rehabilitation plan was developed in collaboration with the user. Subsequent follow-ups, 3–5 days a week for about 4–6 weeks, involved home visits with training interventions targeted at the user’s specific goals, including outdoor activities in outdoor places. In the development of the outdoor reablement model, a Place Attachment Assessment Tool (PAAT) [20] was also developed to support a patient-centered approach to outdoor engagement in reablement practice. The assessment tool provided a framework for identifying the individual’s unique attachment to outdoor places. By building on, and facilitating personal motivation for outdoor engagement, the assessment tool formed a basis for goal-setting practice, which involved outdoor activities. Detailed descriptions of the tool, and a manual for conduction can be found in the original publication [20].

Given that outdoor initiatives may be limited by a range of overlapping and interrelated factors, Curry and colleagues [36] recommend considering elements at the individual, interpersonal, and contextual levels when designing outdoor recreation programs. The implementation of new interventions presents significant challenges and often fails because of a lack of adaptation to the specific context [37, 38]. Therefore, a ‘one size fits all’ model for outdoor reablement is inadequate because contextual adaptations that consider environmental factors are necessary. Proctor and colleagues [39] developed a taxonomy to theorize various aspects of successful implementation. Domains such as acceptability, adoption, appropriateness, costs, feasibility, fidelity, penetration, and sustainability all influence the success of an implementation. In this study, we focus particularly on the concepts of appropriateness, an intervention’s suitability and relevance for a given setting, and feasibility, whether the intervention can be successfully executed within that setting. The aim of this study was to tailor an outdoor reablement model that is appropriate and feasible for the unique context in rural Arctic Norway.

## Methods

This action research study is part of a larger project that aimed to create an outdoor reablement model. A fundamental assumption in action research is that scientific knowledge is socially constructed through collective reflections and that the planning, execution, dissemination, and implementation of research are not separate actions but are deeply interconnected [40, 41]. In a recent publication [20], we described how reablement teams can promote outdoor activities for reablement participants on the basis of a goal-centered assessment exploring different facets of place attachment. In this paper, we aim to ensure the appropriateness and feasibility of implementation, as we tailor the model to a rural Arctic setting in Norway. The study design is built on the experience based co-design (EBCD) methodology [42], which emphasize a user-centered bottom-up process for service design, building on a strategy with roots within the paradigm of social innovation [43, 44]. This approach was chosen to secure an experienced-based and democratic approach and to facilitate implementation, which is described to strengthen the acceptability of new interventions [39]. User involvement is argued to be particularly crucial when designing and planning for interventions that aim to promote active participation among older people [8]. In accordance with the EBCD study design, we utilized a multi-stakeholder design to highlight contextualized experiences, which are important for sustainable implementation of services [43, 45] and enhance creative problem-solving and innovation [46–48].

### Study setting and participants

Participants from three municipalities in northern Norway were invited to participate in the study. The municipalities were strategically selected and two of them had experiences with offering outdoor activities as part of the reablement service. Municipality 3 had recently established a political and organizational strategy focused on preventive and health-promoting services to support aging in place. Consequently, particular attention was directed toward services that could enhance independency and functional abilities of older people living in ordinary housing. Actors from this municipality were involved in the development of the design and aim of the study. Thorough anchoring and user involvement are known to ensure the high quality and relevance of the research [49].

All three municipalities are situated in rural Arctic areas characterized by scattered settlements with limited public transportation opportunities and are located far from larger cities in northern Norway. These areas are exposed to harsh weather for large parts of the year. The climate is characterized by a winter period that includes snow and ice from November to April. During mid-winter, including the polar night, there are few hours of daylight. The summer period stretches from late May to late August and includes a period of midnight sun, i.e., daylight 24 h per day at most.

Staff from reablement services, including physiotherapists (PTs), occupational therapists (OTs), registered nurses (RNs), and associated nurses (ANs) from the respective municipalities, were invited to participate in the study. Additionally, a diverse array of stakeholders from various healthcare facilities and health administration sectors within Municipality 3 (Table 1) were involved. All stakeholders, except two, had a minimum of a bachelor’s degree in health profession, and three were leaders in public health services. Building on a user-centered perspective, five members from the local senior council and three older people who had participated in reablement services were invited to participate in the study. This varied group, with its range of backgrounds and geographical affiliations, was strategically chosen to offer a broad spectrum of contextualized experiences, which are considered critical for the development of sustainable service designs [43, 45].

Ultimately, 35 stakeholders (eight men) volunteered to participate in the study. Further details regarding the municipalities and participants involved can be found in Table 1.

**Table 1:** Participant information

Municipalities	Inhabitants	Geographical size		
**Municipality 1**	2 500	812 km2		
**Municipality 2**	9 700	480 km2		
**Municipality 3**	11 400	424 km2		
**Participants**				**Number of participants**
** Reablement staff**	3 Physiotherapists (PT) 3 Occupational therapists (OT) 1 Registered nurse (RN) 1 associate nurse (AN)		*N* = 8	
** Additional stakeholders**	16 stakeholders from healthcare facilities and health administration			*N* = 16
** User participants**	5 representatives from the local senior council 3 reablement participants (RP)		*N* = 8	
** Research team**	2 PTs 1 RN		*N* = 3	
**Total number of participants**			*N* = 35	

### Data generation

The study took place from August 2020 to June 2021, and data were collected through an approach that included (1) a co-design workshop, (2) focus groups, (3) individual interviews, and (4) a second co-design workshop. Throughout the data generation, the focus was on features (individual, organizational, and environmental) that participants considered important for an outdoor reablement model in a rural Arctic setting while acknowledging that the components included in the model should be appropriate and feasible for implementation [39].

Data collection occurred amidst the COVID-19 pandemic, a period marked by restrictions on intermunicipal travel, necessitating a hybrid approach to our methods. Activities were carried out within Municipality 3, where the majority of participants were based and could attend physically. Moreover, stakeholders from other municipalities engaged in workshops and focus groups digitally.

#### Stage 1: Co-design workshop 1

We organized a one-day workshop (Table 2) that brought all participants together to share experiences and collaboratively develop ideas for tailoring outdoor reablement to a rural Arctic setting. The workshop commenced with “trigger presentations” that showcased experiences from a health facility providing outdoor activities in similar settings. Researchers did also share literature on the potential benefits, effects, and challenges of outdoor activities. These presentations were utilized to facilitate a shared understanding of the project’s goals and to stimulate innovative thinking and idea generation [42, 50].

**Table 2:** Program for workshop 1

9:00–11:00 am	Introduction with “trigger presentations”
11:00–12:30 am	Three separate co-design groups
12:30 − 1:30 pm	Break
1:30 − 3:00 pm	Plenary discussions and summing up

Following the presentations, the participants were divided into three co-design groups to delve into specific central themes:

- Group 1 focused on recruitment strategies and goal-setting approaches.
- Group 2 discussed the use of aids and equipment.
- Group 3 considered seasonal adaptations and safety measures.

Each co-design group was provided with individual thematic guides tailored to their respective topics. We also encouraged spontaneous reflections and ideas. The action researchers (ME, BAS, SH) acted as facilitators in the group discussions, with a co-mediator responsible for taking notes. Eliassen and colleagues [50] have argued that action researchers are particularly well-positioned to facilitate co-design processes due to their neutral stance when engaging with various stakeholders who represent differing and potentially conflicting perspectives. By ensuring that every voice is represented in the discussions and by highlighting both similarities and differences in the perspectives that emerge, action researchers can facilitate in-depth discussions in co-design processes [50]. We utilized the trigger presentations as a starting point for discussions, in line with Donetto and colleagues [42], and elaborated on the participants experiences with the varied themes that represented the groups.

The participants in each group were strategically chosen on the basis of their professional expertise and administrative roles. All the groups included members from the local senior council and featured a diverse mix of stakeholders.

After a lunch break, we reconvened all the participants for a plenary discussion. Each group shared summaries of their discussions, and cross-group feedback was actively encouraged.

On the basis of earlier results, a preliminary outdoor reablement model was created [20], and the participating reablement teams were encouraged to include outdoor activities during a period of six months to gain experience with the outdoor reablement model in the respective municipality. This included an assessment of prioritized outdoor activities, an assessment of the users’ functional capacity, goal setting, and training on outdoor activities and other goal based activities for a minimum of 3 interventions per week for 4–6 weeks (the model is described elsewhere [20]).

#### Stage 2: focus groups

Approximately two months after the workshop, we conducted three digital focus groups with the reablement teams from the respective municipalities (*N* = 8). Data from workshop 1 informed the semistructured thematic guide (Appendix), which was developed in line with descriptions by Polit and Beck [51]. The purpose of the focus groups was to generate thorough descriptions of experiences with both success and challenges from conducting outdoor activities as part of reablement and to gather knowledge about contextual features that could be potential facilitators of or barriers to outdoor reablement. The focus groups were led by one of the authors (BAS), and another author participated as a co-mediator (ME). The focus groups lasted for approximately 60–70 min and were audiotaped and transcribed.

#### Stage 3: individual interviews with reablement participants

As the reablement teams in the respective municipalities were encouraged to initiate outdoor activities, we recruited three of the involved RPs for individual interviews. RPs that had been involved with outdoor activities during their reablament interventions were contacted by the reablement team members. The inclusion criteria were an age of 65 years or older, included in a reablement program, and an interest in and ability to perform some form of outdoor activity. We developed an interview guide (Appendix) based on tentative analysis of currently generated data from the project, allowing the reablement participants to elaborate on their experiences with the outdoor reablement model. The questions evolved around experiences with outdoor activities, both currently and through a life-course perspective. Connectiveness with nature and places were central. We explored the experiences with interventions in the project and posed questions related to both the challenges and the perceived benefits of the outdoor interventions. Furthermore, we asked questions specifically related to the potentials and challenges of different seasons in the Arctic context and attempted to capture experiences within the unique Arctic environment. The interviews were carried out physically at the participants home after the participant had been engaging in outdoor reablement for a minimum of three weeks. Each interview lasted approximately 60 min. The interviews were audiotaped and further transcribed.

#### Stage 4: workshop 2

Six months after the first workshop, we invited the participants to a second digital workshop. This one-day workshop included a presentation of the tentative results and the preliminary model for outdoor reablement in a rural Arctic setting [20]. A plenary discussion was held to allow for input and adjustments from all participants. Notes were taken during the workshop, and data collected from this event were used to refine the model further through subsequent analyses.

**Fig. 1:**
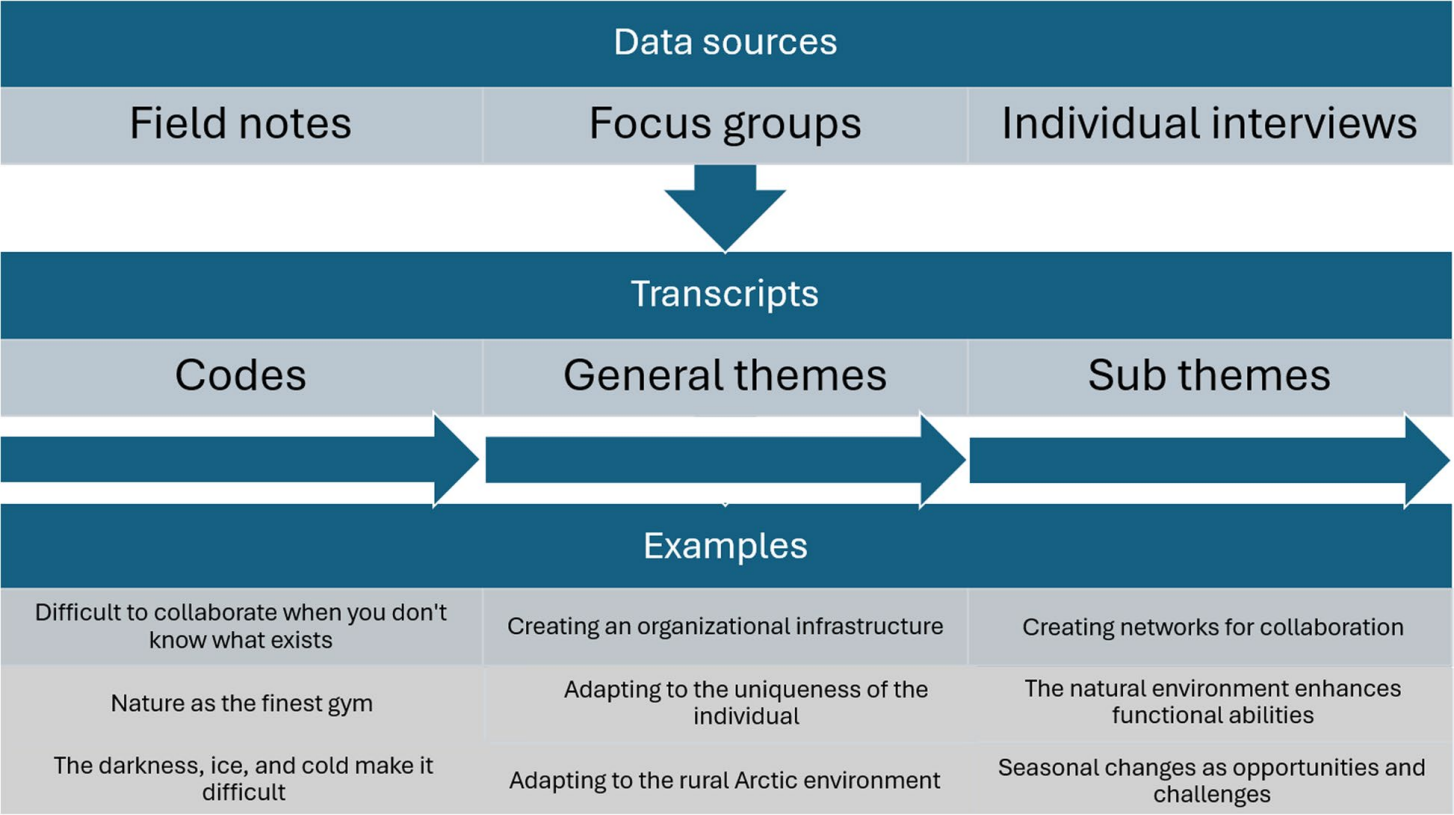
*Analysis approach of merged data sources*,* exemplified by codes and themes*

In summary, the data in this project included notes from plenary discussions and co-design groups in the workshops, transcriptions from three focus groups with reablement teams and three individual interviews with RPs (Fig. 1).

### Analysis

The framework of reflexive thematic analyses by Braun and Clarke [52] structured the analysis, which allowed for a systematic, though fluid, approach to coding and theme development. First, all data (field notes from workshops, audio recordings from focus groups and interviews) were transcribed into written text. At this stage and further on, all data were handled jointly, creating codes that linked the diverse sources together (Fig. 1). After the data were read multiple times, we identified meaningful units that were relevant for the study aim, which were coded and further systematically categorized. The coding process was in line with principles of constructive research approaches [53], as we created codes that were direct wordings from the original text material, allowing for an initial inductive approach, highlighting the voice of the participants. Despite adopting an inductive approach to exploring meaning, we cannot overlook the study’s focus on the appropriateness and feasibility of the model, which may have influenced how we interpreted the results in light of this premise. This focus have shaped our analytical lens, guiding our understanding of the results.

Consecutively, we opened for a continuously iterative movement between data and theory, in line with the abductive research approach [52, 53], allowing for a theoretical interference with the data. Consecutively, we constructed general themes based on patterns of shared meaning across the diverse data sources and theory. Further on, we reviewed and refined the themes and structured them into subthemes. Fig. 1, presents an example of how initial codes were transformed into the main themes, and further categorized into subthemes.

During the coding process, we became aware of the multiple factors at different levels that were relevant for adapting the model to an Arctic rural setting. To cope with this complexity, we framed our analysis within a socioecological perspective, which embraces the interrelations between people and their environments [54–56]. The socioecological perspective draws on system theories and has been emphasized as a framework in the development of health care interventions [54, 57] and aligns with the ideologies of integrated care as an interrelation between the micro (individual), meso (organizational), and macro (environmental) levels of service delivery [58]. A central aspect of the socioecological perspective is that it combines both individual and environmental aspects, which makes it suitable for serving as a metamodel in the development of appropriate and feasible health care services [59, 60]. We have utilized the framework of micro, meso, and macro levels to structure the results in this study.

## Results

**Fig. 2:**
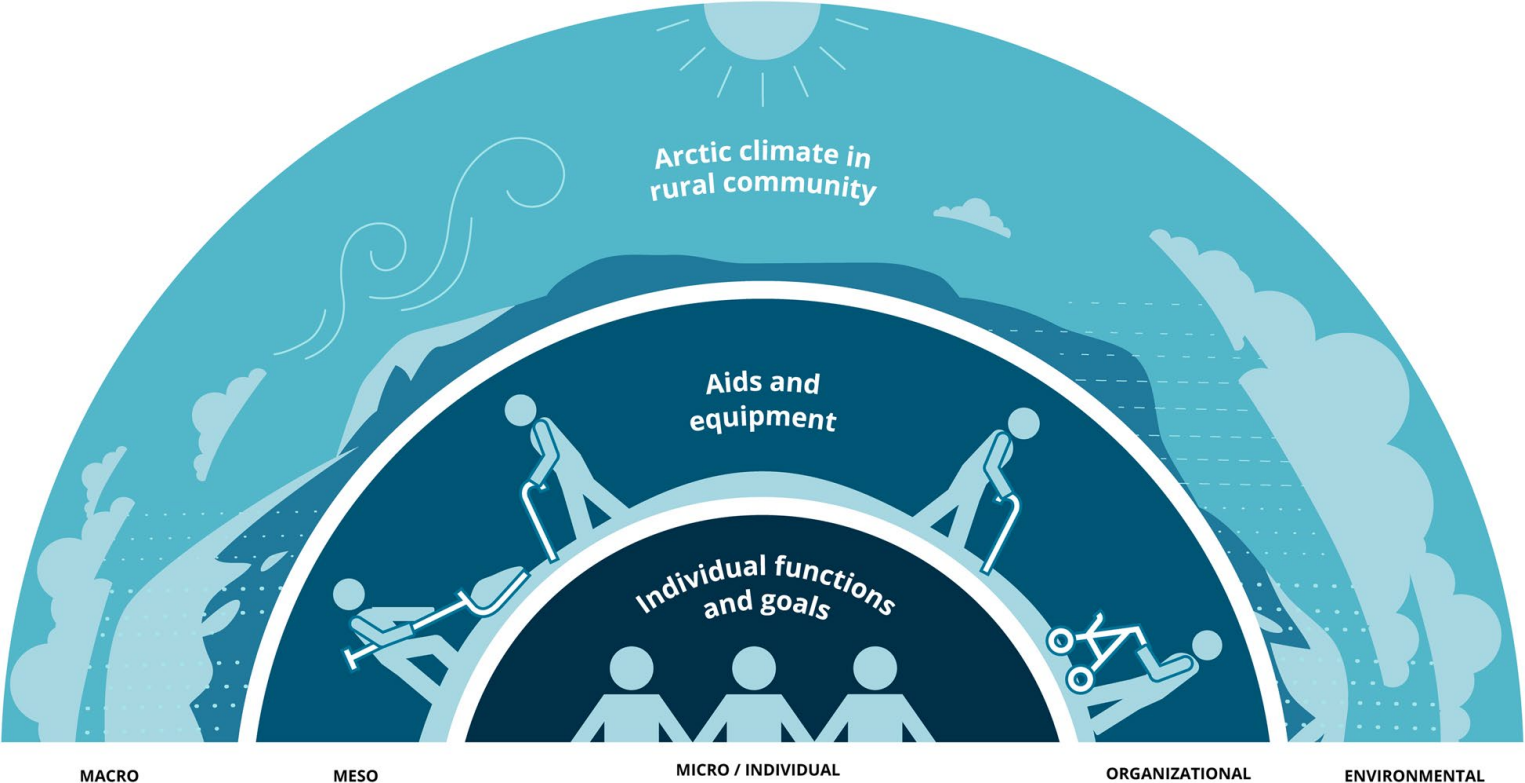
A socioecological model for outdoor reablement in a rural Arctic setting inspired by the ‘rainbow model’ by Valentijn et al. [61]

Through the analysis, we identified multiple features on respectively the micro, meso, and macro level, constituting the three themes: (1) Adapting to the uniqueness of the individual, (2) Creating an organizational infrastructure, and (3) Adapting to the rural Arctic environment. These features present both challenges and opportunities for incorporating outdoor activities into reablement in this setting. The results contribute to a socioecological model that reflects the complexities of conducting outdoor reablement in a rural Arctic setting (Fig. 2).

### Adapting to the uniqueness of the individual

#### The natural environment enhances functional abilities

The participants highlighted a range of potential advantages associated with engaging in outdoor activities in the Arctic environment. Moreover, they endorsed the integration of outdoor spaces into reablement services, citing the benefits to physical and mental functioning. One of the PTs suggested that snow could serve as a natural ‘balance pad’, which would be beneficial for functional training:

PT3: *“Moving around in the snow can provide even better balance training than training on a conventional balance pad inside”*.

The natural environment was lauded as an optimal setting for physical exercise, offering inherent challenges that could enhance balance, strength, and endurance. Engaging in outdoor activities was viewed as a complex action that inherently facilitated a variety of integrated training elements, which could yield significant benefits for an individual’s functional improvement. A reablement participant noted that nature is “*the finest gym there is”*, and the quote below reveals how the natural environment withholds “hidden” elements of exercise:

RN1: “*There’s something genius about being outdoors: If you think about having to go down 9 steps to get to the garden*,* and then there’s cobblestone for a bit*,* and then a curb to get over into the garden*,* and then a hilly garden where you might want to kneel down and add some bark or something like that. Those are quite complicated exercises for someone who has just managed to get across the kitchen floor and into the bedroom. Therefore*,* from my point of view*,* it is just win‒win‒win because you can practice a lot… some people love workout routines and exercises*,* but I think we will meet most of those who… if you can sneak those exercises into concrete tasks… I mean activities…”*.

The participants also emphasized the significance of outdoor settings for social and mental well-being, as one PT stated,

PT3: *“Getting them outside the home*,* I believe it gives them something extra. I think that being out in the fresh air releases extra endorphins […]. I do not think we have ever had anyone who went out for a walk and came back in unhappy about having gone out or thinking that it was a bad experience*,* quite the opposite”*.

Although increasing physical and mental functions were key arguments for outdoor activities, the discussions also prominently featured the richness of connecting activities to individualized goals and meaningfulness.

#### Supporting individual meaning making

The discussions underscore the importance of personal inclination toward outdoor activities when implementing individualized reablement plans. Engaging in nature would be perceived as fundamental for many people in Northern Norway (engagement in practical chores, recreation, and social participation); however, not everyone would find meaning in outdoor engagements. Understanding each participant’s unique connection to and desire for outdoor engagement was thus a critical component of goal setting within the reablement team. A deep-rooted attachment for nature was seen as a potential catalyst for outdoor activities:

RN1: *“In [my community]*,* most individuals have some history with nature; be it through harvesting or simply enjoying a walk… They’re connected to their local surroundings*,* familiar with every hill and inlet that they can view from their window”*.

The significance of places outside of people’s residences was discussed by both the reablement staff and the reablement participants, which implied the unique person‒environment interplay in meaning making. Emotions and experiences related to nature, weather, seasonal changes, and sensory stimuli were highly valued:

RP2: *“Getting out in nature…. The first thing you experience is the fresh air*,* and then you have nature all around. You can just sit down and watch it; you’ll never get tired of it. The birds that sing in the springtime*,* the thawing snow… I can see how things are changing in nature. I like to fish*,* and considering the condition that I am in now*,* I will go out fishing this summer and pick cloudberries in the mountains”*.

Capturing what is meaningful for each person was nevertheless discussed as being challenging. A social worker pointed out,

Social worker: *“Many have quit doing activities they were previously engaged in. One must delve into each person’s motivation*,* asking ‘What is important to you?’ Indeed*,* the answer to this question is not always readily apparent”*.

This highlights the complexities involved in tailoring interventions to meet the unique needs and interests of individuals, emphasizing the importance of understanding their personal motivations and values.

### Adapting an organizational infrastructure

For the organization to support the delivery of outdoor activities within a rural Arctic setting, available aids and equipment and multi-actor collaboration were identified as key features.

#### The organization needs to have available aids and equipment

The challenges of engaging older people in Arctic outdoor activities were widely recognized, particularly the risks associated with winter conditions, including ice and snow on staircases at entrances and walkways. Proper footwear was identified as a crucial preventive measure against outdoor hazards:

OT2: *“Footwear is a key preventive factor. The market offers various effective ice creepers [rubber band devices with ice spikes for shoes]”*.

There was a clear agreement that the reablement team should have access to aids and equipment to address both individual and environmental challenges. The aids discussed during Workshop 1 and subsequently acquired by the team are detailed in Table 3.

**Table 3:** List of aids and equipment the project team purchased

Walking sticks
Ice creepers [rubber band device with ice spikes for shoes]
Umbrella crutch [an umbrella that could be used as a crutch]
Walking stick and walker for outside use
Berry picker with extension rod
Seating stick [a walking stick that easily could be transformed to a seat for resting]
Folding cane
Warm clothing to the reablement team [warm waterproof and windproof jackets and pants]

The requirements for diverse aids and equipment varied significantly among individuals, necessitating a collaborative relationship with the local assistive technology center, which provided a selection of necessary aids for the user. During the group discussion in Workshop 1, a representative from the assistive technology center shared that there are numerous aids available and that healthcare professionals could improve their outreach to discover what options might be available, stating,

Assistive technology manager: *“One must collaborate with each other. It is crucial to achieve a dialog with the assistive technology center in the municipality*,* as there is much to gain here”*.

In response, one RN remarked,

RN2: *It’s difficult to collaborate when you are unaware of the available options. Few people know what they can apply for or obtain. There are many assistive devices that are not well-known”.*

These reflections highlight a need for increased communication and collaboration between healthcare personnel and assistive technology managers.

However, some environmental modifications fell outside the scope of the reablement team’s responsibilities, which required collaboration with a much broader scope of stakeholders within the community and would demand networks for collaboration.

#### Creating networks for collaboration

Adequate street lighting was recognized as crucial for visibility during the dark winter months, which can persist even at midday in Arctic regions. The issue of icy and slippery streets and stairs was also raised, with the team relying on community efforts to apply sand or grit for safety. The availability of resting places and benches during outdoor activities was another concern:

OT1: *“We have encountered situations where there is nowhere to rest during walks. We have experienced the need for amenities such as benches”*.

Addressing these challenges called for a broader network of collaboration including stakeholders from the political and administrative levels of the municipality to develop solutions that ensure age-friendly communities.

Furthermore, the social worker called for enhanced partnership with local organizations and providers of other community activities, such as exercise groups or recreational associations. Such partnerships were deemed crucial for challenging the rigid boundaries of the health care system, promoting innovative thinking and new structural solutions to enhance opportunities for outdoor recreation. One challenge, however, was to obtain a good overview of various actors in the community. A general practitioner (GP) pointed out that although this could be a central challenge, smaller communities may be better positioned to handle such challenges. As she mentioned,

GP: *“Not everyone has a complete overview of the different actors in the municipality. It’s important to have knowledge of each other and each other’s expertise. […] But in smaller municipalities like [our municipality]*,* it’s easier to know about each other*”.

### Adapting to the rural Arctic environment

The diversity of the Arctic climate and its seasonal fluctuations enables a broad spectrum of activities and sensory experiences. Nonetheless, extreme seasonal shifts posed challenges and could act as barriers to outdoor activities. Evaluating the environmental features as potential enablers or obstacles was thus deemed essential for adapting the outdoor reablement model to the rural Arctic setting.

#### Seasonal changes as opportunities and challenges

The various seasons presented both opportunities and distinct challenges that were important to consider in the adaptation of an outdoor reablement program.

The transition from winter to summer during the spring results in significant changes, with temperatures rapidly rising and the longer daylight hours, blossoming flora, and general revival of nature make this period ideal for outdoor pursuits. Gardening and maintenance tasks, accessible even to those confined to their immediate surroundings, were highlighted as valuable activities for people living in these areas. The sensory experience of nature’s transformation was also highly appreciated. Consecutively, the summer season was viewed as an opportune time for outdoor engagement. The warmer temperatures reduced the need for heavy clothing and increased the accessibility of outdoor areas, including those with walking aids, making this season perfect for outdoor activities. One of the reablement participants planned various activities during this season:

RP1: *“I will be gardening*,* fishing*,* berry picking*,* sawing wood*,* mowing the lawn*,* and planting potatoes. It gives me a sense of purpose and tasks to maintain throughout the summer”*.

However, the participants characterized the northern Norwegian summer as brief, with primary healthcare services often reduced due to vacations, which made this season a potential risk phase, due to lack of follow up. Thus, while the summer season provided good opportunities for outdoor activities, a lack of available resources for reablement was seen as a sub-optimal situation to make optimal use of these opportunities.

Autumn was described as a season conducive to recreational and sensory experiences in nature. Crisp air, colorful foliage, and the harvest season that traditionally hold significance for many. However, erratic weather, characterized by rain and wind, was considered a barrier, and appropriate clothing was seen as a necessity.

The long winter, spanning from October to April, is marked by extended darkness due to the absence of the sun above the horizon for a mid-winter period. However, phenomena such as “blue hours” and northern lights (aurora borealis) were cited as potential motivators for outdoor activities. The snowy landscape was considered both a facilitator and a potential barrier for outdoor engagement. The population in these areas generally had a sense of identity related to winter activities such as skiing, ice skating, and kicksledding, which are also common manual means of short-distance transportation in the Arctic. However, snow and ice were also described to increase the risk of falling. In that sense, the winter season was also identified as a risk phase for outdoor activities.

OT1: *“We avoid taking risks. Even with ice spikes*,* there is a sense of insecurity. The cold also plays a part. We tend to facilitate more outdoor activities in the spring and summer”*.

Under extreme conditions, the team would even discourage outdoor activities for safety reasons.

Despite these challenges, the participants agreed that while the environment might limit outdoor activities, it did not restrict the incorporation of nature experiences indoors. They suggested enjoying nature through a window at home or from a car. In one municipality, they offered a digital cycling program that combined video footage of familiar landscapes with an indoor exercise bike, providing an immersive experience outdoors. The potential of technology to enhance nature experiences warrants further investigation.

#### A flexible organization adapted to the rural community

The rural setting implied that some of the recipients lived far from the health care facilities, limiting the accessibility of societal services. Additionally, a long travel time is required for the reablement team. One participant stated that the amount of time consumed was a barrier. However, not all people would require the same time resource, and in that matter, they could adjust the time spent traveling due to a flexible way of organizing the service:

OT1: *“A flexible organization is crucial. By quickly identifying who might need more time and who is ready upon our arrival. It is important that our presence does not feel rushed to them”*.

A flexible approach was deemed essential to accommodate varying time requirements and unexpected events:

PT2: *“Our workdays are unpredictable*,* so we maintain a high degree of flexibility. Given that our clients are often frail*,* cancellations and schedule changes are common. We adapt and plan one day at a time”*.

The necessity of transportation emerged as a critical factor to consider. The expansive geographic distances within the community, in combination with limited public transportation, could reduce access to community facilities and activity offers in the local community. This issue would require the reablement team to achieve an overview of existing transportation opportunities that could be relevant to the reablement participants.

## Discussion

Based on a multistakeholder perspective, we developed a socioecological model that accounts for individual, organizational, and environmental conditions for outdoor activities for older persons, aiming to strengthen the models’ appropriateness and feasibility for implementation in a rural Arctic setting.

When developing aging in place strategies in a rural Arctic setting, a holistic approach that accounts for a broad view of contextual factors is needed as the effectiveness of complex interventions is critically influenced by the setting in which they are implemented [28, 38, 62]. The most evident result of this study is knowledge about how several adaptations are needed when tailoring an outdoor reablement model to a rural Arctic setting.

### Person-centered approach

Our results imply that it is imperative to tailor interventions based on a person-centered approach that responds to the individual’s physical and mental capabilities, personal goals, and sense of meaning, in line with core principles in reablement [31, 32]. Building on meaningful outdoor activities is particularly pertinent in a rural Arctic setting, where the population has traditionally lived in close harmony with nature [14]. In accordance with McCormarck and McCance [63], working with a person’s beliefs and values is one of the fundamental principles of a person-centered approach, in addition to shared decision-making, witch is a prerequisite for developing personalized goals. Assessing prioritized outdoor activities, functional capacity, and place attachment is a central element of the Place Attachment Assessment Tool (PAAT), which was developed particularly for the outdoor reablement model [20]. The pursuit of personalized goals related to outdoor activities seems to ensure the appropriateness of the model since such activities may be deeply intertwined with a person’s strong connection to the land, seasons and places, as well as reflecting a cultural heritage related to the unique Arctic context [20]. By considering the individual’s history and cultural background, reablement practitioners can facilitate activities that resonate with the individual’s affective, cognitive, conative, and social place attachment. However, as stated by McCormack and McCance [63], accomplishing such person-centered approaches require a set of prerequisites by the practitioner, i.e. professional competence, interpersonal skills, and commitment to the job. Hence, the attributes of the practitioners will affect the feasibility of implementation. McCormack and McCance [63] also state that the ability to deliver person-centered care is heavily influenced by the care environment in which the approach is delivered. The study’s results add to the understanding of how places are fundamental physical, social, and cultural and, by that, gather people and the environment into one arena of common engagement [21, 64]. The integration of natural environments in reablement is a feasible component of the model by providing a dynamic ‘gym’ that offers a variety of opportunities for physical exercise, sensory stimuli, social interaction, and personal fulfillment.

Moreover, the rural Arctic setting provides a unique backdrop for outdoor reablement, where traditional activities such as fishing, berry picking, gardening, and observing wildlife are aligned with perceptions of meaningful well-living [14], as well as establishing a feeling of being independent. In this sense, outdoor activities may not only promote physical health but also contribute to mental well-being and independence by fostering a sense of identity and continuity with past lifestyles. Sánchez-González and Egea-Jiménez [8] argue that active aging is strongly influenced by each person’s unique life story and a sense of belonging. Living in harmony with nature has been crucial for survival and good health in areas in Northern Norway. Historically, those who mastered the art of harvesting from both sea and land have thrived the most. Many people who are at older age today, grew up during times when the harshness of life and scarcity of food was a central part of life.

### Appropriate and feasible for the rural setting

Individual variations concerning the RPs’ functional level, in addition to varied travel distances in the community, call for a flexible service structure that enables adjustments during the workday. This is in line with research on reablement practices in general [65–67]. Mjøsund and colleagues [68] reported that time consumption was a barrier for engaging in outdoor activities in reablement. A flexible organization is therefore a prerequisite for succeeding with outdoor reablement.

The results indicate the importance of collaborating with stakeholders at a political and administrative level in the municipality to arrange for benches, streetlights, and transportation. Others have also reported that a lack of facilities such as toilets and benches may constrain older people from engaging in outdoor contexts [36], which strengthens our assumption of the value of collaboration with stakeholders responsible for such facilities in the municipality. In accordance with McCormack and McCance [63], an appropriate skill-mix and supportive organizational systems that commit to enhance quality of care, is essential to achieve person-centered approaches. Collaboration, particularly teamwork, has been a central characteristic of reablement [31], but descriptions have been limited to involving collaboration within the reablement team [69–72]. However, descriptions of intersectoral collaborations with stakeholders external to the reablement team are lacking, indicating an area for potential development. The results also revealed uncertainty about who had the mandate to initiate changes or who was responsible for establishing such collaborations. The finding that the termination of the reablement period can be a risk situation for maintenance of progressions made, underscore the necessity for further exploration of how new forms of collaboration could be established and implemented.

Arranging services that account for potential barriers in the community aligns with several of the eight domains within the Age-Friendly Cities and Communities (AFCC) framework [73]. The AFCC network was established by the World Health Organization in 2010 to connect cities, communities and organizations with the aim of promoting active and healthy aging [62, 73]. While reablement is part of the domain *community support and health services*, we have shown how an outdoor reablement model also relates to the domains of *social participation*,* outdoor spaces*, transportation, and *respect and social inclusion.* Whereas AFCC studies have traditionally focused on urban areas [9, 74], we argue that outdoor reablement services may be an appropriate initiative for promoting age-friendliness in a rural Arctic setting.

### Appropriate and feasible for the Arctic setting

Adapting to climatic variations and environmental challenges and facilitators were highlighted as particularly important for outdoor reablement in this rural Arctic setting and related to both the appropriateness and feasibility of the model. All the seasons were described as containing motivational aspects that could be harnessed to promote outdoor activities, as seasonal changes were discussed as a source of motivation for active participation and fostering hope for future engagement.

However, our study also identified potential constraints, such as inclement weather, slippery conditions, and limited daylight. Slippery surfaces in the immediate surroundings of dwellings are a significant concern for the safety of participants and are associated with a high risk of falls. Similar concerns about the fear of falling potentially restricting older people from engaging in outdoor activities have been noted by others [68, 75]. Currie and colleagues [36] also highlighted that feeling unsafe outdoors can be a barrier to outdoor engagement. In their study, Arnadottir and colleagues [76] compared activity levels between individuals in Arctic rural and urban areas and reported that those from rural areas had a greater risk of falling. The harsh climate in the Arctic threatens the feasibility of implementing outdoor reablement. According to Sánchez-González and Egea-Jiménez [8], security is a main pillar of active aging, as a sense of safety may promote independence and physical activity among older people. To mitigate this threat, our model includes assessing the need for aids and equipment that could support older adults and prevent falls. This adds to our earlier descriptions of assessments of outdoor reablement [20], where we developed the Place Attachment Assessment Tool (PAAT), which support assessment of prioritized outdoor activities and functional ability at the individual level. The results of the present study show how environmental aspects should also be included in the assessment. Nonetheless, there is a need to monitor potential fall incidents in future evaluations of the model.

The winter period in the Arctic limited outdoor engagement due to adverse weather conditions, which have been identified by others as a significant barrier to outdoor activities among older people [36]. While harsh conditions may dampen the motivation for outdoor activities, it is necessary to explore how to prevent this extended period from leading to functional decline, which could affect the ability to engage in activities during springtime. Some of the participants from the reablement teams had experiences with a digital bicycling program to simulate outdoor activities during periods when outdoor engagement was perceived as unsafe. The potential of virtual environments to compensate for seasonal constraints is a promising area for further research. In their randomized controlled trial, Rendon and colleagues [77] reported that virtual reality gaming tools improved dynamic balance and balance confidence in older people. The Safe step application designed to support fall prevention exercise has been shown to be feasible [78, 79] and results in promising effects on reduced rates of falls [80]. However, further evaluations are needed to assess the potential of integrating virtual outdoor initiatives into reablement programs.

Spring and summer were notably preferred for engaging in outdoor activities. Consequently, we recommend an increased emphasis on outdoor reablement during the spring. However, during the summer, reablement activities may be limited due to vacation periods, which could have implications for feasibility as well as equitable reach of reablement. Therefore, it is crucial to involve other stakeholders who can provide activities or engage family members when service delivery activity is low. Many older people in rural Arctic areas have younger family members who have moved away to more urban areas. This results in many having less robust social networks. However, during the summer, it is not unusual for family and acquaintances to take the time to visit their relatives, thereby supporting activities during this period. Ensuring social networks that support active aging aligns with the fundamental aspects of reablement [31].

### Aging in (a meaningful) place

In this study, we conceptualize aging in place as something more than aging in the person’s home but emphasize the close interplay between people and places. Furthermore, we consider place of residence to include not only the home but also outdoor areas that constitute meaning for a person. Our results show how an outdoor reablement model has the potential to facilitate aging in place by targeting the intersection between individual, social and contextual features between older people and places, in line with strategies for age-friendly environments displayed in the AFCC framework [73].

Traditionally, the perspectives of functional decline and frailty in older people have been associated with the physically frail body rather than the interplay between the person and the environment in which they engage, which constitute their identity and meaning of life [81]. Bjerkmo and colleagues [11] argue that coping with everyday life requires not only individual capacity but also adaptations of the environment. Currie and colleagues [36] argue that an understanding of the relationships among nature, aging and the physical environment is fundamental in the development of age-friendly communities and the enhancement of older people’s engagement in outdoor environments. The interplay between the person and the physical and social environment is therefore crucial when designing aging in place initiatives such as outdoor reablement. To reflect the highly varied and individual interpretations of significant places, we advocate for a broader interpretation of the concept of Aging in place that transcends older people’s home to encompass the entire community. We propose that aging in place should also involve reconnection with outdoor spaces that hold personal significance for the individual, proposing the concept of *Aging in meaningful places*.

Whereas our results indicate that the model has the potential to support people in reengaging with outdoor activities, we acknowledge that there are inherent risks that may limit the appropriateness and feasibility of such interventions across diverse settings. In this study, we have identified critical risk phases that may hamper implementation, such as the winter period with its challenging climate, the summer vacation with staff on holiday, and the period post-reablement when follow-up by professionals ends. These critical phases need to be addressed to support the sustainable and long-term effects of outdoor reablement interventions, which are also important domains for implementation outcomes, in accordance with Proctor and colleagues [39].

### Strengths and limitations

A strength of this study is the use of a co-design methodology to acknowledge the importance of contextual adaptations [82], where researchers closely collaborate with older people and local stakeholders to tailor an outdoor reablement model to be appropriate and feasible for adoption in rural Arctic settings. The inclusion of multiple perspectives and voices enhances the democratic validity of the research [83] and ensures its direct practical relevance [40, 43]. However, the results must be interpreted acknowledging that the participants in the study were initially positive and enthusiastic about implementing this type of intervention.

Features of the model were tested in a limited period of time (six-months)with a small number of service recipients, the acceptability, adoption, and long-term uptake [39] of the model, including information about the target population reached (Re-aim), remains to be explored. In addition to addressing implementation outcomes, the effectiveness and cost-effectiveness of the model need to be addressed in future evaluations.

Although the co-design methodology can be considered a strength, it calls for reflection about the involved participants. The participants represent the health-care administration of municipalities, whereas other sectors in the municipality, e.g., those responsible for transportation and accessible outdoor environments, were not included. In light of aging populations and the complexity of the demographic challenge, a system-thinking approach that includes a broader representation of participants could have been beneficial and should be explored further.

The project spanned from November 2020 to May 2021, a period that allowed the reablement team to trial outdoor activities across different seasons, yielding rich contextual data.

Nevertheless, several aspects of the project merit rigorous discussion. The workshops took place during the COVID-19 pandemic, and social interaction restrictions necessitated a hybrid approach. While most stakeholders participated in person, some had to participate digitally. This arrangement may have constrained the nonrestricted dialog. However, service delivery was not affected by pandemic restrictions and proceeded normally throughout the project.

The involved reablement teams include a small number of reablement staff. Consequently, the number of participants in the focus groups was limited, which may have influenced the depth of the group discussions and limited comprehensive reflections. Only three service recipients who tested outdoor activities were interviewed. Given the small number of participants, we cannot offer any analysis of outcome effects.

## Conclusions

In this article, we presented a socioecological model tailored to enhancing outdoor reablement within a rural Arctic setting. Our work extends the scope of reablement beyond the confines of indoor environments, integrating the outdoor setting as a vital component.

Our findings illustrate the potential of the rural Arctic community to facilitate engagement in meaningful activities and places, leveraging the diverse opportunities presented by the local culture, nature and climate. Nonetheless, we have also highlighted the necessity of adapting to challenges that could impede outdoor engagement and increase the risk of incidents, such as falls.

This model prioritizes individual needs while being sensitive to environmental factors, recognizing the profound role of outdoor spaces in the lives of people living in the rural Arctic, and aims to capitalize on this relationship for therapeutic purposes. With respect to the implementation outcome framework of Proctor and colleagues [39], the results show how the appropriateness and feasibility of the model was ensured through a co-design model that emphasized local experiences and first-hand knowledge.

## Supplementary Information

Supplementary Material 1.

## Data Availability

Due to the qualitative nature of the data in this study, and to protect participant confidentiality, as participants could be identified within the raw data, the full transcripts are not public available. However, anonymized excerpts supporting the findings are included within the article. Additional de-identified data are available from the corresponding author upon reasonable request.

## Abbreviations

AFCC: Age-Friendly Cities and Communities
AN: Associate nurse
GP: General practitioner
OT: Occupational therapists
PT: Physiotherapists
PAAT: Place Attachment Assessment Tool
RFF: Fund of Nordland
RN: Registered nurse
RP: Reablement participant
