# Supplementary material for: Aging in (a meaningful) place – appropriateness and feasibility of Outdoor Reablement in a rural Arctic setting
Source: BMC Health Serv Res. 2024 Dec 18;24:1580. doi: 10.1186/s12913-024-12031-7 (PMC11653717; doi:10.1186/s12913-024-12031-7)
Supplement: Supplementary file 1 — Supplementary Material 1. [file 12913_2024_12031_MOESM1_ESM.docx]

**Appendix**

**Focus group guides**

| Themes | Guiding questions | Follow-up questions |
| --- | --- | --- |
| Introduction and creating confidence | Introducing the project, project aim and setting |  |
|  | Can you tell about yourself; professional and educational background, experiences with reablement services and outdoor recreation? |  |
|  | What connection do you personally have to outdoor recreation? |  |
| Organizational settings in the reablement services | How does a workday normally look like? |  |
|  | Your team started practicing reablement services for only a short while ago. How do you experience the start up? | How did you share tasks and responsibility?  What kind of assessment tools are used in your reablement practice?  Who is the typical reablement participant? |
| Experiences with outdoor activities in reablement settings | What was the initial rationale behind implementing outdoor activities? | Why did you consent to participate in this project? |
|  | What was your experiences of the workshop and what processes was generated within the reablement team after participating in the workshop? | What formal and non-formal procedures and practices were generated?  How were tasks and responsibility shared and distributed? |
|  | How did you recruit participants, and how did they respond to the service offer? | How did you introduce outdoor activities to them?  Did they have to be motivated to go outside? Can you elaborate on challenges if you experience any? |
|  | Can you provide us with some positive experiences or cases of particular interest? | Follow up to get thorough descriptions about cases: Who were the user, goals, motivation, places of relevance, social network of relevance? |
|  | Can you provide us with some less positive experiences that you have had? | Follow up to assess possible challenges: Physical, motivational, social, cultural or environmental challenges? |
| Recruitment and goalsetting | What kind of people would you say fit for outdoor recreation offerings in reablement? |  |
|  | What assessment guides the goalsetting practice? | What is important to assess? How can you contribute to motivate participants? |
|  | What do the participants perceive as motivating factors for engaging in outdoor recreation? |  |
| Arctic cultural factors | Why do you think this population finds outdoor recreation to be of significance? |  |
|  | Can you try to describe any cases that you have experiences that reveal a cultural connection between people and the nature in which they live in? |  |
| Arctic environmental factors | Have you experienced any challenges concerning weather, climate, or other possible issues? |  |
|  | How do you plan for challenges in the outdoor climate during seasonal changes? | Equipment, tools, clothing, aids? |

**Individual interviews**

| Introduction and creating confidence | Can you tell about how you got in contact with the reablement team in the first place? | Story behind engagement in the reablement service?  Had you heard about the service? What were your expectation, goals, concerns, etc.? |
| --- | --- | --- |
| Outdoor activities as part of reablement services | How did you experience being introduced to outdoor activities by the reablement team? |  |
|  | Can you tell about the details of the outdoor interventions? | What was done? By whom? Where did you go? Was the place and activities familiar to you? |
|  | How did you and the reablement staff talk about goals and preferences? | Were there used any standardized tools? Free conversation? Leading questions or suggested activities by the team? |
|  | Can you tell about the outdoor activities that you perceive as of highest significance for you? | Why do you think these activities are important for you? |
| Experiences with outdoor activities | What activities do you normally do outside? |  |
|  | What places are of importance to you? |  |
|  | What motivates you to be outside in nature (or other outdoor areas)? |  |
|  | Can you tell about your connection with being outdoor in nature? | Can you tell about earlier outdoor experiences of relevance from childhood up to this current data? |
|  | Have you had any connection with outdoor activities based on your work or leisure activities? |  |
| Arctic environmental factors | Are there any seasons that you are more outside than others? How do seasonal changes affect your engagement with outdoor activities? |  |
